# Diversity of Bacterial Communities on Four Frequently Used Surfaces in a Large Brazilian Teaching Hospital

**DOI:** 10.3390/ijerph13020152

**Published:** 2016-01-22

**Authors:** Tairacan Augusto Pereira da Fonseca, Rodrigo Pessôa, Alvina Clara Felix, Sabri Saeed Sanabani

**Affiliations:** 1 Clinical Laboratory, Department of Pathology, LIM 03, Hospital das Clínicas (HC), School of Medicine, University of São Paulo, São Paulo 05403 000, Brazil; tairacanaugusto@hotmail.com (T.A.P.F.); rodrigo_pessoa1@hotmail.com (R.P.); 2 São Paulo Institute of Tropical Medicine, University of São Paulo, São Paulo 05403 000, Brazil; clarafelixx@gmail.com

**Keywords:** bacteria, microbiome, hospital surfaces

## Abstract

Frequently used hand-touch surfaces in hospital settings have been implicated as a vehicle of microbial transmission. In this study, we aimed to investigate the overall bacterial population on four frequently used surfaces using a culture-independent Illumina massively parallel sequencing approach of the 16S rRNA genes. Surface samples were collected from four sites, namely elevator buttons (EB), bank machine keyboard buttons (BMKB), restroom surfaces, and the employee biometric time clock system (EBTCS), in a large public and teaching hospital in São Paulo. Taxonomical composition revealed the abundance of Firmicutes phyla, followed by Actinobacteria and Proteobacteria, with a total of 926 bacterial families and 2832 bacterial genera. Moreover, our analysis revealed the presence of some potential pathogenic bacterial genera, including *Salmonella enterica*, *Klebsiella pneumoniae*, and *Staphylococcus aureus*. The presence of these pathogens in frequently used surfaces enhances the risk of exposure to any susceptible individuals. Some of the factors that may contribute to the richness of bacterial diversity on these surfaces are poor personal hygiene and ineffective routine schedules of cleaning, sanitizing, and disinfecting. Strict standards of infection control in hospitals and increased public education about hand hygiene are recommended to decrease the risk of transmission in hospitals among patients.

## 1. Introduction

Infected or colonized patients are likely to shed microbes in hospital environments, leading to nosocomial transmission, which represents an important cause of morbidity and mortality [1,2,3]. It has been estimated that approximately two million patients per year in the United States acquire a nosocomial infection and that at least 90,000 of them succumb and die [4,5,6]. This estimation makes nosocomial infections the fifth leading cause of death in acute-care hospitals [6]. In Canada, a point-prevalence survey reported that 11.6% of adults in hospital experience a health care-associated infection [1]. A recent study by Quach *et al.* [7] indicated that a visit to the emergency department was associated with a more than threefold increased risk of infection. In earlier study undertaken at oncology and neurology units in Brazil, the density of health-care-associated infection were reported to exceed 80 episodes per 1000 patient-days (PMID: 9437483) [8]. Other data pooled from four studies conducted in Brazilian neonatal intensive-care units (PMID: 17433942, 15484803, 11287879, 11852413) [9,10,11,12] revealed an overall incidence of health-care-associated infections of 40.8 infections per 100 patients (95% CI 16.1–71.1) and a density of 30.0 episodes per 1000 patient-days (25.0–35.0). It is known that bacteria can survive on various surfaces including white coats [13], stethoscopes [14], adhesive tape [15], computer keyboards [16], elevator buttons [17], mobile communication devices [18], and ultrasound transducers [19], far longer than previously believed [20]. Most of the bacterial species characterized in the previous studies originate most likely from the normal skin flora such as coagulase-negative staphylococci [16,17,18,21]. The link between human use and the composition of bacterial communities have also been reported on surfaces in kitchens and restrooms with bacterial species originating from human skin flora colonizing on kitchen surfaces, in agreement with frequent skin-to-surface occurrences [22,23,24].

Here, we sought to investigate the diversity and distribution of bacterial contamination on hand-touch surfaces in public areas of a large public and teaching hospital in São Paulo. To this end, we comprehensively characterized the bacterial communities found on a surface of elevator buttons (HC-EB), bank machine keyboard buttons (HC-BMKB), HC-restroom surfaces, and the employee biometric time clock system (HC-EBTCS) using a culture-independent Illumina massively parallel sequencing approach of the 16S rRNA genes*.*

## 2. Experimental Section

Surface samples were collected from four sites (EB, BMKB, restrooms surfaces, and EBTCS) in the Hospital das Clínicas (HC), the largest public hospital in South America with 2200 beds. For the EB, three elevators were selected because they are connected to the majority of patient floors and available to patients, visitors, and healthcare professionals. Six HC-EB surfaces, three exterior buttons, and three interior buttons were sampled. Surfaces of seven HC-BMKB that are commonly used by patients, visitors, and healthcare professionals were also swabbed and included in this study. Samples were also collected from 18 surfaces in three male and three female public restrooms including door handles into and out of the restroom, faucet handles, and toilet flush handles. Finally, six surfaces of HC-EBTCS, three on the first floor, two on the fourth floor and one on the fifth floor were swabbed and included in the study. All surfaces were sampled using sterile swabs moistened with ST solution (0.15 M NaCl and 0.1% Tween 20) [25]. The head of each swab was aseptically cut from the handle and directly placed into bead tubes containing 60 μL of Solution C1 (PowerSoil manufacturer’s 1st lysis solution). To detect possible contamination, a new sterile swab in ST solution was tested as a negative control with each set of test swabs. The DNA from each swab including negative controls was extracted using the PowerSoil DNA kit (MO BIO Laboratories™: Carlsbad, CA, USA) according to the manufacturer’s protocol. The extracted genomic DNA from the same surfaces were pooled together for the subsequent amplification, library preparation, and sequencing. The V4 region of the 16S rRNA gene was amplified using the primers Bakt_341F/Bakt_805R (5′-CCTAC GGGNGGCWGCAG-3′, 5′-GACTACHVGGGTATCTAATCC-3′ [26]. Amplification was performed in two steps using a custom Illumina (San Diego, CA, USA) preparation protocol in which the first PCR was conducted with forward primers that contained partial unique barcodes and partial Illumina adapters. The remaining ends of the Illumina adapters were attached during the second PCR and the barcodes were recombined in silico using paired-end reads as previously described [27]. The amplified products from the second PCR were separated by gel electrophoresis and purified using Freeze N Squeeze DNA Gel Extraction Spin Columns (Bio-Rad: Hercules, CA, USA). Each purified amplicon was quantified on a Qubit 2.0 Fluorometer (Life Technologies: Carlsbad, CA, USA), pooled at equimolar concentration, and diluted to 4 nM. To denature the indexed DNA, 5 µL of the 4 nM library were mixed with 5 µL of 0.2 N fresh NaOH and incubated for five minutes at room temperature. Then, 990 µL of chilled Illumina HT1 buffer were added to the denatured DNA and mixed to make a 20 pM library. After this step, 360 µL of the 20 pM library was multiplexed with 6 µL of 12.5 pM denatured PhiX control to increase sequence diversity and then mixed with 234 µL of chilled HT1 buffer to make a 12 pM sequenceable library. Finally, 600 µL of the prepared library was loaded on an Illumina MiSeq clamshell style cartridge for paired end 300 sequencing. The library was clustered to a density of approximately 820K clusters/mm^2^. Image analysis, base calling, and data quality assessment were performed on the MiSeq instrument (San Diego, CA, USA).

To confirm that the PCR reagents were not the source of bacterial sequences, PCR of the no-template control was performed. Also, prior to extraction and amplification, all reagents and ultrapure water were exposed to UV light of 254 nm for at least three minutes. No visible amplification signal was observed for the no-template control on a gel, indicating that bacterial contamination was minimal.

The library was clustered to a density of approximately 917K clusters/mm^2^. Image analysis, base calling, and data quality assessment were initially performed on the MiSeq instrument. Any reads containing two or more ambiguous nucleotides, low quality scores (average q score < 25), or reads shorter than 300 bp, were discarded. For the 16 s primer trimming, two nucleotide mismatches to the adjacent PCR primer were allowed. MiSeq forward and reverse reads were paired using the PANDAseq v.2.9 [28] with default parameters. Potential chimera sequences were detected and removed using the UCHIME algorithm [29]. To reduce computational burden analysis, 10% of reads were randomly selected from each sample and considered for further analysis. To avoid sampling size effects, the number of reads per sample was normalized to 1837 for each dataset by randomly subsampling to the lowest number of reads among samples. The taxonomic classification of each read was assigned against the EzTaxon-e database [30] at a 97% threshold of pairwise sequence similarity. The richness and diversity of samples were determined by Chao1 estimation and the Shannon diversity index at 3% distance. The bacterial community diversity indices (Shannon estimator) were calculated using Mothur and Shannon-ace-table.pl software programs (Chunlab Inc.: Seoul, Korea). The overall phylogenetic distance between communities was estimated using the Fast UniFrac [31] and visualized using principal coordinate analysis (PCoA). To compare operational taxonomic units (OTUs) between samples, shared OTUs were obtained with the XOR analysis of CL community program v3.43 (Chunlab Inc.: Seoul, Korea). The sequencing data have been uploaded to zenodo [32].

## 3. Results and Discussion

Using the Illumina sequencing-by-synthesis method (MiSeq platform), 4,706,332 reads were generated by the four samples. After filtering, 3,940,871 effective sequences remained, accounting for nearly 83.7% of the total sequences. To minimize computational time, a random 195,814, 791,476, 548,605, 708,726 of reads from the EB, EBTCS, BMKB, and restrooms were computationally selected. This resulted in a total of 2,244,621 valid reads, of which 2,040,614 (90.9%) were derived from bacterial sequences, 202,773 (9.03%) from an eukaryotic source, 527 (0.02%) unmatched sequences, and 97 (0.004%) from Achaea. Analyses were limited to bacterial populations. The unmatched sequences of OTUs failed to be assigned into any genus with a confidence level higher than 50%, suggesting the presence of many novel bacteria. The distribution of sequence lengths produced agreed with the amplicon length (464 bp) of the 16S rRNA. Bacterial sequences ranging from 195,814 to 708,726 were contained in the four sample groups, and the OTU ranged from 78,236 to 315,177 (Table 1). The indices of bacterial diversity were estimated using a rarefaction curve based on OTUs.

This analysis indicated 97% similarity of OTUs at the 3% divergence was attained for each sample and suggests an adequate depth of coverage. By rarefaction analysis estimates, the trend for species richness on different sample groups was quite similar to each other. The Shannon index was calculated to estimate the alpha diversity. The Shannon index computed at 3% dissimilarity showed the lowest value of evenness (9.064) for the sample group HC-EB compared to all the other samples that revealed the highest value of evenness (Table 1).

**Figure 1:**
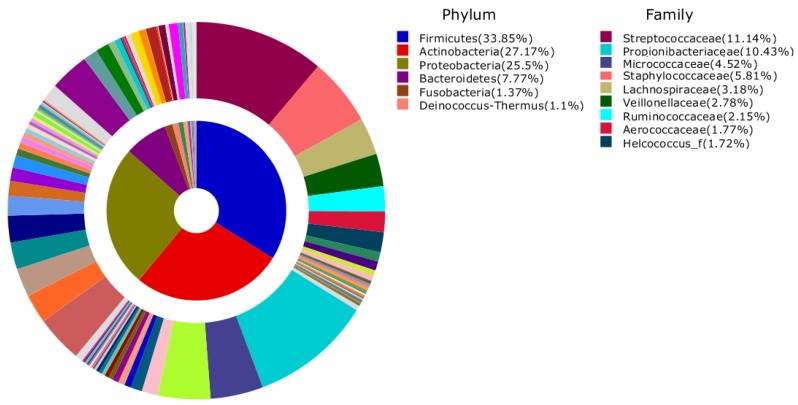
Average composition of bacteria from all samples (inner area: Phylum, outer area: Family). Phyla and Families with more than 1% of their proportion were represented.

**Figure 2:**
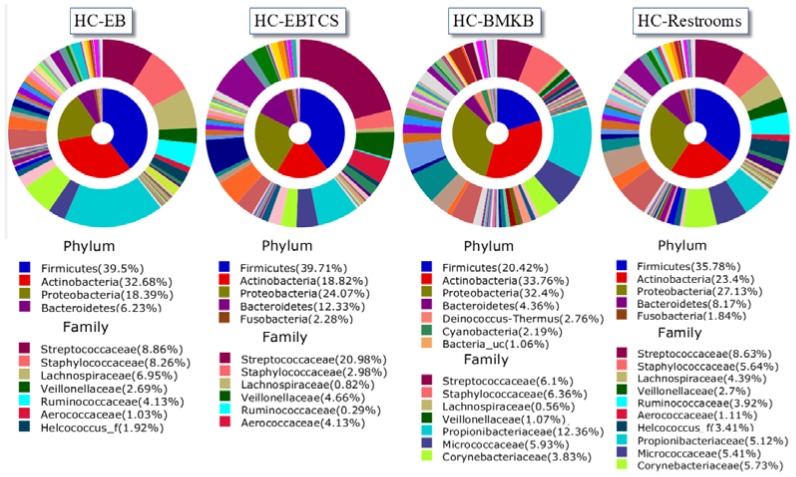
Average composition of bacteria from each sample (inner area: Phylum, outer area: Family). Only bacterial phyla and families that had a relative abundance of 1% or greater are presented.

The bacteria were from 41 phyla, 131 classes, 360 orders, 926 families, and 2832 genera. Firmicutes (33.8%) was the most abundant phylum with 11.1% contributed by Streptococcaceae, 5.8% Staphylococcaceae, and 3.2% by Lachnospiraceae. The most abundant OTUs at phylum and family levels that accounted for more than 1% of all sequences are shown in Figure 1. Firmicutes were commonly most abundant in each sample accounting for 39.5%, 39.7%, and 35.8% in HC-EBTCS, and HC-Restroom, respectively (Figure 2). The microbiota of HC-BMKB consisted mostly of Actinobacteria (33.8%), followed in decreasing order of relative abundance by Proteobacteria (32.4%), Firmicutes (20.4%), and Bacteroidetes (4.4%).

**Table 1:** Library reads and sequence diversity of 16 S rRNA.

Sample ID	Valid Reads	Number of OTU (>97% Identity)	Shannon Index	Goods Library Coverage
HC-EB	195,814	78,236	9.065327	0.645633
HC-EBTCS	791,476	315,177	9.109842	0.61603
HC-BMKB	548,605	238,544	9.801774	0.594236
Hc_Restrooms	708,726	288,265	9.782897	0.615365

**Figure 3:**
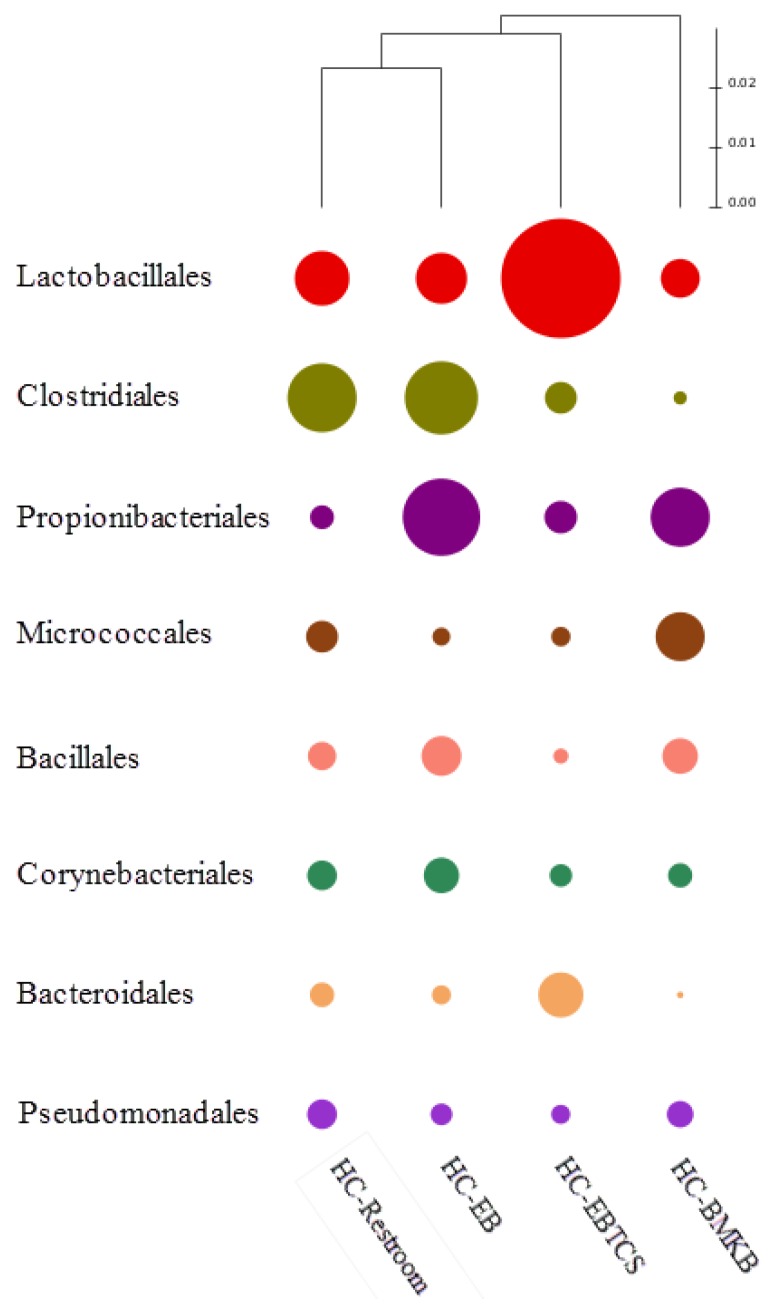
Heat map to compare the bacterial communities between the four samples in terms of Order.

The heat map analysis for the bacterial communities at the order level among the four groups showed that the proportion of *Lactobacillale* in the HC-EBTCS (26.8%) was more than twice greater than on the HC-EB (11.5%) and HC-Restroom (12.3%), and more than three times larger than on the HC-BMKB (8.7%) sample group. On the other hand, second dominant phylum, Clostridiales, were equally abundant in HC-Restroom (15.5%), and HC-EB (16.4%); these were more than twice and three times greater than HC-EBTCS (7.2%) and HC-BMKB (3%) (Figure 3). Compared to other sites sampled in this study, the bacterial population on the surfaces of the HC-BMKB was the most diverse and more diverse than the bacterial communities on the surfaces of HC-Restroom. The six OTUs of the most abundance species associated with the four sample libraries were related to *Propionibacterium acnes* (2.23%–11.8%) and *Streptococcus dentisani* (1.54%–8.45%) (Table 2).

**Table 2:** Identities of the six most abundant OTUs in the bacterial communities.

	HC-EB	HC-BMKB	HC-Restroom	HC-EBTCS
Abundance Order	Taxa (Abundance)			
1	*Propionibacterium acnes* (11.8%)	*Propionibacterium acnes* (7.46%)	*Propionibacterium acnes* (2.23%)	*Streptococcus dentisani* (8.45%)
2	*Streptococcus dentisani* (2.8%)	*Propionibacteriaceae*_uc_s (2.18%)	*Rothia mucilaginosa* (1.76%)	*Propionibacterium acnes* (4.72%)
3	*Staphylococcus epidermidis* (2.54%)	*Streptococcus dentisani* (1.85%)	*Streptococcaceae*_uc_s (1.57%)	*Streptococcaceae*_uc_s (3.84%)
4	*Propionibacteriaceae*_uc_s (2.46%)	*Propionibacterium*_uc (1.56%)	*Streptococcus dentisani* (1.54%)	*Streptococcus*_uc (3.08%)
5	*Propionibacterium*_uc (2.41%)	*Staphylococcus epidermidis* (1.31%)	HQ762034_s (1.34%)	*Streptococcus salivarius* (2.2%)
6	EF188441_s (1.54%)	*Streptococcaceae*_uc_s (1.26%)	*Staphylococcus epidermidis* (1.33%)	*Rothia mucilaginosa* (1.67%)

HC-EB: Elevator button, HC-EBTCS: Employee biometric time clock system, HC-BMKB: Bank machine keyboard buttons.

**Figure 4:**
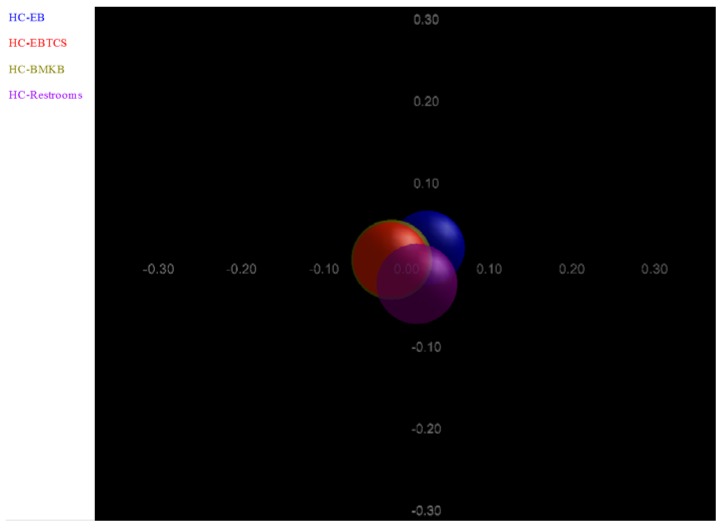
Principal Coordinates Analysis (PCoA) analysis of the microbiome of each surface sample based upon Fast UniFrac method with normalization option. Different colored symbols are indicative of the various surfaces.

The weighted Principal Coordinates Analysis (PCoA) of the microbiome of each sample based upon the UniFrac method was performed to compare overall composition of the bacterial community within the samples. In the two-dimensional plot visualized from the Unifrac weighted distance matrix PCoA, all samples grouped in one cluster with no apparent difference in average size of their circles as depicted in Figure 4.

A large community of microorganisms lives underneath the bright lights and on the stainless steel gurneys and other environmental sites in hospital. Most of these microbes are harmless and are brought to hospital via human bodies. Because humans harbor different types of microbes on different parts of their body [33,34,35] it is likely that different surfaces host different microbial species because of frequent contact. Determining how microbial assemblages colonize in a hospital environment is particularly important to elucidate the main sources of hospital acquired infections, which have long been among the leading causes of patient deaths [36,37].

Here, we explored the deep sequencing analysis of microbial populations associated with some surfaces touched by hands in one of the largest clinical hospitals in Latin America using culture independent Illumina next generation sequencing technology. Our findings revealed that the predominant phyla (in terms of percentages and reads) were *Firmicutes*, *Actinobacteria*, *Proteobacteria*, and *Bacteriodetes*. These results are consistent with previously published studies of microbiota colonizing human skin [35,38]. Similar to our study, culture-based analysis of bacterial colonization of elevator buttons in large teaching hospitals in Toronto, Ontario found that staphylococci, along with streptococci were among the most prevalent bacteria [17]. Also, the prevalence of these phyla has been reported previously in various studies on computer keyboards [16], ultrasound transducers [19], and in a variety of indoor environment surfaces [24,39,40]. The enrichment of the surfaces of the HC-EB and HC-Restroom in *Firmicutes* (*Ruminococcaceae* and *Lachnospiraceae*) and *Bacteroidetes* suggests their fecal contamination because these taxa are generally associated with the human gut [33,41,42]. These results are worrisome from a public health perspective because of the similar way of dissemination of enteropathogenic bacteria and human commensals.

Obviously our DNA study did not provide information on whether the bacteria we observed were cause for concern; pathogenic taxa are thus suspected from previous studies. Within the dominant phyla in this study, the bacterial families with the highest abundance across all samples were Streptococcaceae, Propionibacteriaceae, Micrococcaceae, and Staphylococcaceae. The presence of *Streptococcus* has been frequently reported from surfaces where person to person contact may occur [16,17,23,43]. In the present study, we identified the presence of more than 35 Streptococcus spp. (tentatively, *S. dentisani, S. salivarius, S. sanguinis, S. gordonii, S. lactarius, S. parasanguinis, S., and S. rubneri*) in all surface swab samples. *Streptococcus dentisani*, *Streptococcus rubneri,* and *Streptococcus lactarius* are novel *streptococcus species* and have recently been assigned to the *Streptococcus mitis* group. The members in this group are known as commensal bacteria of the human oral cavity, gastrointestinal tract, and the female genital tract; however, invasive infections might occur when entering the bloodstream [44]. *Streptococcus pyogenes* (also known as group A streptococci), an important human pathogen that causes a variety of diseases in immunocompetent individuals, were observed at low frequency on the surfaces of HC-EB and HC-EBTCS. *Propionibacterium acnes*, a well-described member of the skin microbiome [35] were clearly more abundant on surfaces. A variety of species of *Staphylococcus* including *S. epidermidis, S. haemolyticus, S. hominis, S. pasteuri, S. saprophyticus, and S. aureus* also were observed on all surface samples. Among them, *S. epidermidis* is the most abundant bacterial species, followed by *S. capitis*, *S. hominis,* and *S. saprophyticus*. *S. epidermidis* is the most frequently isolated species from human epithelia [45] and accounts for at least 22% of bloodstream infections in intensive care unit patients in the USA [46]. Furthermore, *S. epidermidis* may be involved in prosthetic joint, vascular graft, surgical site, central nervous system shunt, and cardiac device infections [47]. The formation of biofilm matrix *is a* major virulence factor that protects *S. epidermidis* from both the host immune response and antibiotics [48,49]. We also identified *S. aureus,* which has the capacity to cause a variety of devastating infectious diseases [3,50,51]. Of note, *S. aureus* can survive on surfaces for extended periods of time [52]. Outbreaks and infections caused by *S. aureus* have previously been associated with exposures to different contaminated fomites including, toys, towels, razors, handrails, and whirlpools [53]. Several studies have also reported the transmission of *S. aureus* from public places such as gymnasiums, schools, and athletic facilities [43,54,55,56,57]. Detection of *S. aureus* in this study is of great public health concern especially in hospital settings because colonization by *S. aureus* significantly increases the likelihood of a person developing infections. Further studies are needed to determine the prevalence of antibiotic resistance of *S. aureus* isolates detected in the current study.

Other potentially pathogenic bacteria such as *Salmonella enterica*, *Klebsiella pneumoniae*, *Enterococcus faecalis*, *Pantoea agglomerans, Bartonella_uc, Clostridium perfringens* have been detected in all our sample groups but at low abundance. The presence of *Salmonella enterica* in our study may indicate that hospital users are either exposed or have had prior exposures to the infection source. With all data collectively considered, identification of these bacteria may represent a serious public health hazard.

Our approach to this investigation reports the presence of bacterial populations regardless of whether they are dead or alive, culturable cells, or non-culturable cells. Therefore, future study using RNA-based approaches, such as RNAseq, is needed to confirm the existence of viable bacterial populations on these surfaces. Despite the identification of these bacteria in our samples, it is difficult to trace the source of a community-acquired infection back to these sites. Also, our study suggests that these surfaces may contribute to the transmission of potentially harmful bacteria and should be a matter of concern for public health authorities. Although, alcohol-based hand sanitizers do not eliminate all bacteria or microorganisms, but reduce the number of microbial contamination to levels that are considered safe from a public health standpoint. Also, to keep the bacterial contamination to a minimum, higher compliance with contact precautions along with enhanced surface cleaning could be essential. Gebel and colleagues (PMID: 23967396) [58] have concluded in their recent review that there is a need for defining standard principles for cleaning and disinfection that focus on improving the quality of and the compliance with environmental disinfection procedures. With regard to surface cleaning, the existing cleaning methodologies were clearly evaluated in a recent review by Weber and Rutala and were found to have little effect [59]. Therefore, a technology that limits environmental contamination of infectious microorganisms regardless of human error is urgently needed. Indeed, we need to conduct more studies to better understand the microbial ecosystems in hospitals by investigating how pathogens are transmitted from place to place and from person to person.

## 4. Conclusions

Our study provides a comprehensive assessment of the diversity in bacterial communities and the presence of potential bacterial pathogens on hand-touch surfaces in public areas of hospitals that are frequently used. As reported in the present study, a high degree of microbial diversity derived from these surfaces may be alarmingly attributed to poor personal hygiene. Reductions in these contaminations translate into public health benefits by reducing the rate of hospital acquired infections. To conclude, it is very important to highlight the need for strict hand hygiene, other contact precautions, and regular and enhanced disinfection of environmental surfaces for maximally reducing the spread of disease-causing pathogens.
